# Functional Study of the Hap4-Like Genes Suggests That the Key Regulators of Carbon Metabolism *HAP4* and Oxidative Stress Response *YAP1* in Yeast Diverged from a Common Ancestor

**DOI:** 10.1371/journal.pone.0112263

**Published:** 2014-12-05

**Authors:** Nataliya Petryk, You-Fang Zhou, Kateryna Sybirna, Marie-Hélène Mucchielli, Bernard Guiard, Wei-Guo Bao, Oleh V. Stasyk, Olena G. Stasyk, Olena S. Krasovska, Karine Budin, Nancie Reymond, Sandrine Imbeaud, Sophie Coudouel, Hervé Delacroix, Andriy Sibirny, Monique Bolotin-Fukuhara

**Affiliations:** 1 Institut de Génétique et Microbiologie, IFR Génome 115, Université Paris-Sud and CNRS, Orsay, France; 2 Institute of Cell Biology, National Academy of Sciences, Lviv, Ukraine; 3 Gif/Orsay DNA MicroArray Platform, Gif sur Yvette, France; 4 Centre de Génétique Moléculaire, CNRS, Gif sur Yvette, France; 5 Department of Biochemistry, Ivan Franko Lviv National University, Lviv, Ukraine; 6 University of Rzeszow, Rzeszow, Poland; Texas A&M University, United States of America

## Abstract

The transcriptional regulator *HAP4*, induced by respiratory substrates, is involved in the balance between fermentation and respiration in *S. cerevisiae*. We identified putative orthologues of the Hap4 protein in all ascomycetes, based only on a conserved sixteen amino acid-long motif. In addition to this motif, some of these proteins contain a DNA-binding motif of the bZIP type, while being nonetheless globally highly divergent. The genome of the yeast *Hansenula polymorpha* contains two *HAP4*-like genes encoding the protein HpHap4-A which, like ScHap4, is devoid of a bZIP motif, and HpHap4-B which contains it. This species has been chosen for a detailed examination of their respective properties. Based mostly on global gene expression studies performed in the *S. cerevisiae HAP4* disruption mutant (*ScΔhap4*), we show here that HpHap4-A is functionally equivalent to ScHap4, whereas HpHap4-B is not. Moreover *HpHAP4-B* is able to complement the H_2_O_2_ hypersensitivity of the *ScYap1* deletant, *YAP1* being, in *S. cerevisiae*, the main regulator of oxidative stress. Finally, a transcriptomic analysis performed in the *ScΔyap1* strain overexpressing *HpHAP4-B* shows that HpHap4-B acts both on oxidative stress response and carbohydrate metabolism in a manner different from both ScYap1 and ScHap4. Deletion of these two genes in their natural host, *H. polymorpha*, confirms that *HpHAP4-A* participates in the control of the fermentation/respiration balance, while *HpHAP4-B* is involved in oxidative stress since its deletion leads to hypersensitivity to H_2_O_2_. These data, placed in an evolutionary context, raise new questions concerning the evolution of the *HAP4* transcriptional regulation function and suggest that Yap1 and Hap4 have diverged from a unique regulatory protein in the fungal ancestor.

## Introduction

Evolution of transcription factors and their regulatory networks are particularly interesting to study in the Hemiascomycetes. The phylogenetic distances between species within the phylum are equivalent to the evolution of chordates, and the number of available complete genome sequences and large-scale gene expression data sets is the largest among eukaryotes, and increasing. Moreover this phylum includes *S. cerevisiae*, arguably the most studied eukaryotic organism ([1], [2]). The ecology of yeast species is diverse and these organisms have developed various strategies to compete for nutrient sources and to adapt to stress conditions. While *S. cerevisiae* is a predominantly fermentative species, most yeasts do not possess such a strong fermentative capacity and are respiratory-fermentative, being able to both respire and ferment in different proportions. Respiratory metabolism is also strongly linked to iron metabolism and redox state maintenance.

The expression of *S. cerevisiae* genes is finely regulated by the hierarchy of about 200 interplaying and subordinated transcriptional regulators. Global regulators modulate gene expression profiles according to environmental requirements acting directly on promoters of regulated genes, as well as by communication via other transactivators.

One of these key global regulators in *S. cerevisiae* is Hap4, the transcriptional activator moiety of the CCAAT-binding HAP complex [3] controlling the fermentation/respiration switch [4] [5] [6].

**Figure 1:**
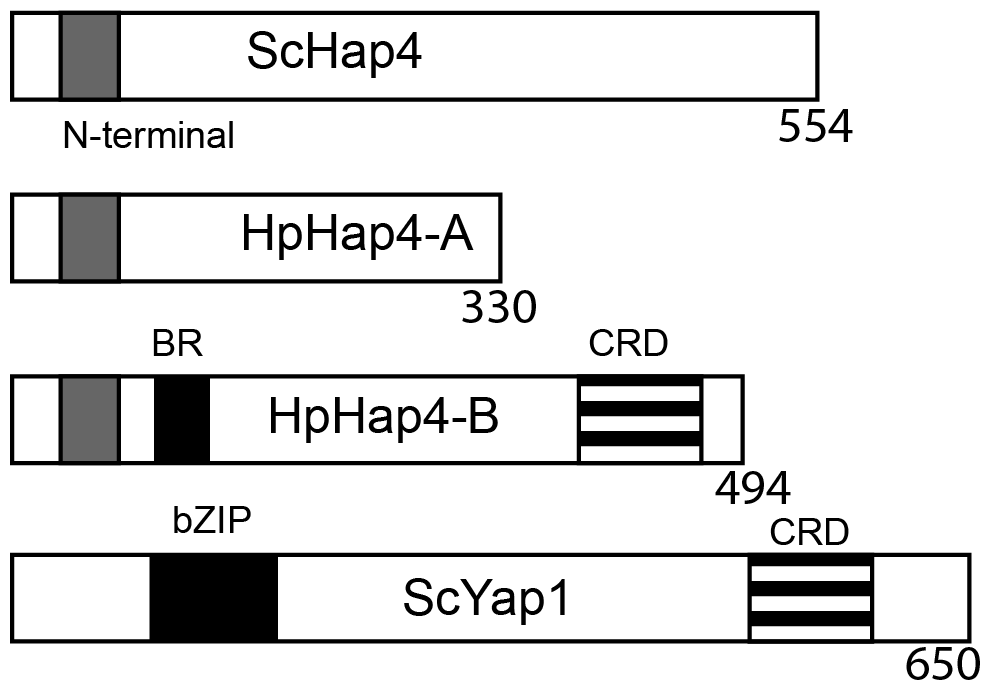
A schematic representation of the Hap4 and Yap1 proteins. The *Saccharomyces cerevisiae* Hap4 and Yap1 proteins are schematically represented relative to the two *Hansenula polymorpha* HpHap4-A and HpHap4-B ones. The main motifs are indicated in grey (N-terminal Hap4), black (bZIP of BR motif; the BR motif is the DNA binding part of the bZIP motif) and striped (CRD or cysteine rich domain).

*HAP2*, *HAP3* and *HAP5* (encoding the core DNA-binding elements of the complex), are highly conserved in all eukaryotes ([7], reviewed in [8] and [9]), but this is not the case for *HAP4*, the activator component of the HAP complex which was originally identified only in *S. cerevisiae* and later in *Kluyveromyces lactis*. These two proteins share a 16 aminoacid-long N-terminal motif [10]. Based on this motif, called here (N-Hap4), we have found putative orthologues in other ascomycetes [11] and discerned two different subclasses of Hap4 proteins. One subclass (identified mostly among species distant from *S. cerevisiae*) contains an additional DNA-binding motif which is the basic region (BR) of the bZIP motif (basic leucine zipper; Prosite PS50217) [12]. BZIP motifs are shared by a family of transcriptional regulators whose archetype is *S. cerevisiae* Yap1, a schematic representation of which is provided Fig 1. Factors which have this BR domain also have a cysteine rich domain (CRD), present in Yap1.

**Figure 2:**
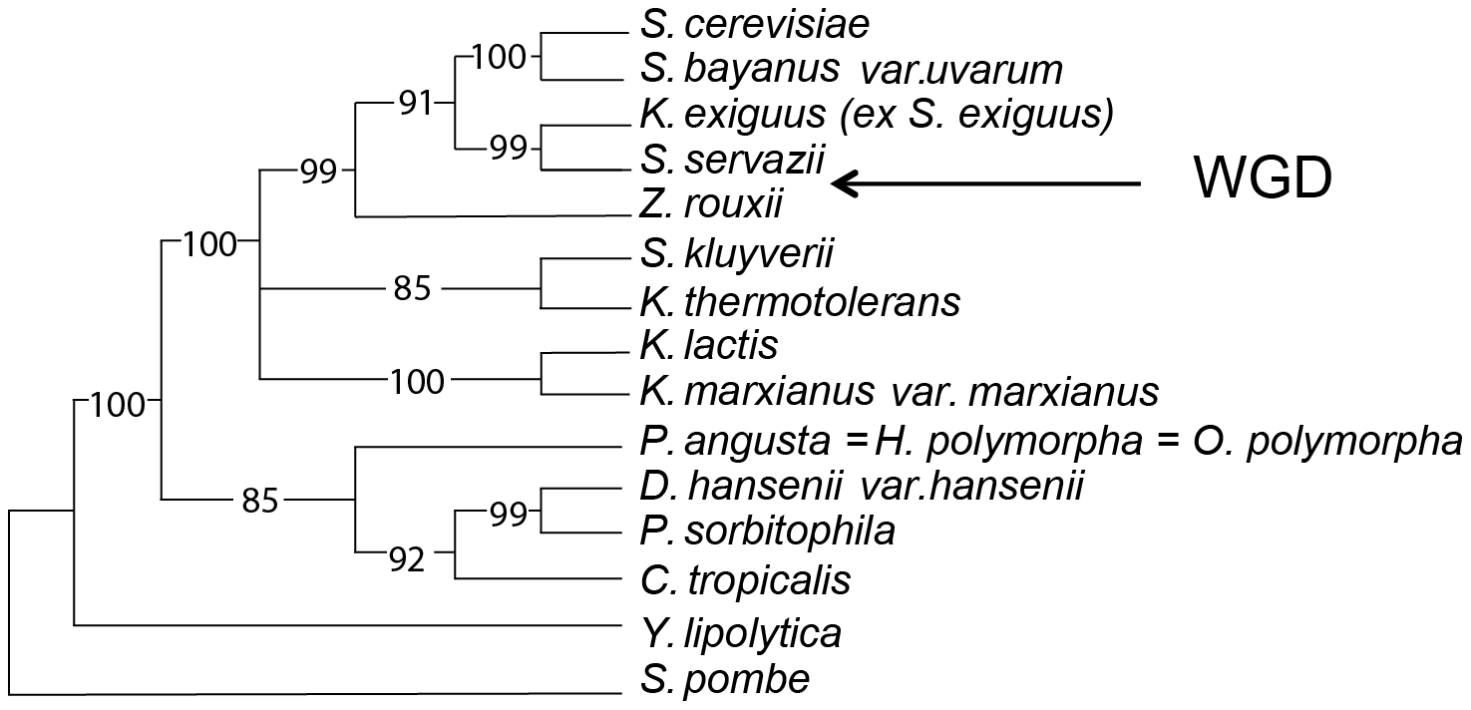
A simple phylogenetic tree of the hemiascomycete yeasts. Only a limited number of yeast species are presented which span the complete clade of Hemiascomycete yeasts from *S. cerevisiae* to *Y. lipolytica, S. pombe* being the outgroup. *H. polymorpha* is placed on the tree at the same position as *P. angusta* which is the anamorphic species. The position of the « Whole Genome Duplication » (WGD) is indicated. The tree is taken from [48].

This prompted us to undertake a detailed study of these two Hap4 types (with and without the bZIP motif) in the yeast *Hansenula polymorpha* (*Taxonomy ID:* 870730). Phylogenetically, this is the species closest to *S. cerevisiae* which contains both Hap4 types. The yeast *Hansenula polymorpha* is well studied. It possesses unique physiological characteristics (thermo-tolerance, ability to utilise various carbon sources including methanol [13]), and is the model organism to study peroxisome functions [14]. It also presents interesting biotechnological properties for the production of heterologous proteins [15] and the degradation of lignocellulose [16]. Its position in a simplified phylogenetic tree is shown in Fig 2.

We previously demonstrated that *H. polymorpha* HpHap4-A, containing only the N-Hap4 motif, is functionally similar to the *S. cerevisiae* protein (ScHap4). It restores the *ScΔhap4* mutant growth on respiratory substrates, is able to interact with the HAP complex and induces the expression of the Hap4 archetype target *CYC1* [17]. We also identified a transactivation domain, different from what was known in *S. cerevisiae* [11]. Later, we showed that the second Hap4-like protein, HpHap4-B, containing the additional bZIP motif, could act in the same way but to a lesser extent, and that the bZIP motif did not contribute to this ability [12].

A range of Hap4-like proteins were recently reported to participate as repressors in iron homeostasis networks. In *Schizosaccharomyces pombe*, Php4 (*PHP4* is an orthologue of *ScHAP4* and does not contain a bZIP motif) acts in iron deficiency conditions as a repressor of genes encoding iron-containing proteins via interactions with the PHP complex (orthologous to ScHAP complex) [18]. *PHP4* itself is tightly regulated at transcriptional [19] and post-translational [20] levels according to iron availability. In *Aspergillus nidulans*, the Hap4-like protein HapX negatively regulates the components of the iron-dependent pathways under iron starvation via its HAP complex [21].

*Candida albicans* genome contains four different *HAP4*-like genes. Hap43, the only bZIP containing Hap4-like protein in *C. albicans*, acts as a transcriptional repressor during iron starvation [22].

Similarly, in *H. polymorpha* the expression of *HpHAP4-B* is also induced in conditions of iron chelation and the disruptant strain is sensitive to iron deficiency [12].

This study aims to distinguish between the two types of Hap4-like proteins. Since the *H. polymorpha* genome sequence was not publicly available when we started this work, we expressed *HpHAP4-A* and *HpHAP4-B* in a *S. cerevisiae* background. Meanwhile, the genome of another *H. polymorpha* strain was sequenced and is now in open access (http://genome.jgi-psf.org/Hanpo1/Hanpo1.home.html).

In this heterologous system, we clearly observed differences between the two Hap4-like proteins. Moreover, we found that HpHap4-B was also involved in the oxidative stress response, similarly to Yap1. These findings, and the analysis of the presence/absence of the two different motifs in the HAP4-like family of transcriptional factors related to their place in the fungi phylogenetic tree, lead us to propose an evolutionary scenario which is further discussed.

## Materials and Methods

### Strains and growth conditions

The yeast strains and plasmids used in this study are listed in Table 1. *S. cerevisiae* strains were grown as previously described [11]. *H. polymorpha* strains were grown at 37°C in YPD medium (1% yeast extract, 2% peptone, and 1% glucose) or in minimal medium (0.17% w/v yeast nitrogen base without amino acids (Difco) with 0.5% w/v ammonium sulphate as a nitrogen source). Amino acids were added to a final concentration of 50 µg/ml as required. For solid media, agar was added to 2% (w/v) final concentration. Cultivation of *Escherichia coli* DH5α and standard recombinant DNA techniques were performed essentially as described [23].

**Table 1:** Strains and plasmids used in this study.

Strain or plasmid	Genotype or description	Reference or source
***S. cerevisiae***		
W303-1A	*MATa ade 2-1 his3-11 leu2-3, 112 trp1-1 ura3-1*	[51]
ScΔ*hap4*	*W303-1A Δhap4::kan*	This laboratory
ScΔ*yap1*	*MATα ade2-1 his3-11 leu2-3,112 trp1-1 ura 3-1 can-100 yap1::HIS3*	[52]
***H. polymorpha***		
NCYC 495 *leu1_1*	*leu1_1*	[53]
NCYC 495 *leu1_1* (pYT1)	*leu1-1:HpLEU2*	[12]
HpΔ*hap4-A*	*hap4-A*Δ*::ScLEU2*	This study
HpΔ*hap4-B*	*hap4-B*Δ::*ZeoR*	[12]
HpΔ*hap4-A*Δ*hap4-B*	*hap4-A*Δ*::ScLEU2 hap4-B*Δ::*ZeoR*	This study
**Plasmids**		
pBFG1	2 µm *LEU2 pPGK1*::3*HA*	[54]
pBFG1-HpHAP4-A	*HpHAP4-A* in pBFG1	[11]
pBFG1-HpHAP4-B	*HpHAP4-B* in pBFG1	[12]
pBFG1-HpHAP4-B-bZip	*HpHAP4-B* with deleted BR motif in pBFG1	[12]
pBFG1-ScYAP1	*ScYAP1* in pBFG1	[12]
pBFG1-ScHAP4	*ScHAP4* in pBFG1	[11]
pYT1	Complements *H. polymorpha leu1_1*	[55]
pHap4-A::ScLEU2	pYT1 H*pHAP4-A::ScLEU2*	This study
pPICZ-B	*Pichia pastoris* expression vector	Invitrogen
pHap4-B::ZeoR	pPICZ H*pHAP4-B*::ZeoR	[12]

### Growth sensitivity tests

Dilutions of overnight YPD cultures were plated on minimal glucose medium (W0) with antimycin A (1 µg/ml) or H_2_O_2_ (0.5 mM) or minimal xylose medium with 5 mM salicylhydroxamic acid (SHAM) supplemented with necessary amino acids and grown at 37°C for 2 days.

### Gel shift experiment

DNA probes were prepared by PCR amplification and end-labelled with polynucleotide kinase and [γ-^32^P]-ATP. Oligonucleotides used to generate the ARE sequence are P17 (5′-CGACGGCTGCCATTAGTCAGCATGGCGCGCAC-3′) and P18 (5′-GTGCGCGCCATGCTGACTAATGGCAGCCGTCG-3′). Analysis of DNA-binding complexes was performed as previously described by [24].

### Construction of the *H. polymorpha Δhap4* deletion mutants

*H. polymorpha ΔHphap4-A* and *double ΔHphap4-A ΔHphap4-B* deletion mutants were constructed by the gene replacement method with NCYC495 *leu1-1* as the parental strain.

A *HpHAP4-A* deletion cassette was constructed in two steps. First, the *HpHAP4-A* upstream-flanking fragment of 1,305 bps ending 3 nucleotides upstream of the ATG start codon was isolated by PCR reaction using primers P9 (5′-TGTGGATCCTTCGAACACAAAGCCTAT-3′) and P10 (5′-GGTTCTAGATCATGGAACCCATTGAAT-3′) and *H. polymorpha* genomic DNA as a template. PCR products were cloned as *Bam*HI-*Xba*I fragments into plasmid pYT1. This produced the intermediate plasmid pHAP4-A-5′. As a second step, the *HpHAP4-A* 3′ region of 1,377 bps starting at nucleotide 379 of *HpHAP4-A* ORF was isolated by PCR with primers P11 (5′-TAACTgCAggTgTCCgACCTgAAAAAT-3′) and P12 (5′-TGGAAGCTTTGAATCCATCGTATAACG-3′) and cloned as a *Pst*I-*Hind*III fragment into plasmid pHAP4-A-5′, producing plasmid pHAP4-A::ScLEU2. This latter plasmid harbours a deletion cassette on which the *HpHAP4-A* region, coding aminoacids 1-126, is replaced with the *S. cerevisiae LEU2* gene. This deletion cassette was excised with *Bam*HI and *Hind*III and transformed in the *leu1-1* recipient strain.

A *HpHAP4-B* deletion cassette was constructed in an analogous manner using the positive selection marker of zeocin resistance [12].

The double knockout strain (*HpΔhap4-HpΔhap4-B*) was obtained by transformation of *HpΔhap4-A* with the *HpHAP4-B* deletion cassette.

*H. polymorpha* NCYC495 *leu1-1* or the derivative prototrophic strain (transformed with pYT1 plasmid) were used as wild-type controls throughout this study as indicated.

### Microarrays

RNA extraction was performed as described previously by [25] and purified using the RNeasy Kit (Qiagen). All cultures were performed on minimal medium plus galactose (2%) and harvested at OD_600_ = 0.8–1. For oxidative stress conditions, 0.5 mM H_2_O_2_ were added in the media for 1 hour. These conditions were chosen in order to compare with previous studies of the *HAP4* and *YAP1* genes of *S. cerevisiae* [5], [26].

RNAs (four independent preparations each time) were prepared from ten different genetic backgrounds in two conditions, with and without oxidative stress (H_2_O_2_): *ScΔhap4* and *ScΔyap1*, each containing the empty plasmid BFG1 as a reference (abbreviated as BFG1/BFG1.H_2_0_2_), BFG1 with the *ScHAP4* gene (ScHap4/ScHap4.H202), BFG1 carrying the *HpHAP4-A* gene (HpHap4-A/HpHap4-A.H_2_0_2_), BFG1 carrying the *HpHAP4-B* gene (HpHap4-B/HpHap4-B.H_2_O_2_). The integrity of the total RNA was determined using an Agilent Technologies 2100 Bioanalyzer and the RNA 6000 Lab-Chip kit. Total RNA concentration was determined using a NanoDrop ND-1000 spectrophotometer [27].

For hybridization, Agilent Yeast oligomicroarrays (v2, cat # G4140B, Agilent Technologies, Palo Alto, CA) were used. Target preparation, hybridization and washing were performed according to the manufacturer's instructions.

The slides were scanned using GenePix 4000B scanner at 100% laser power and the PMT voltage was automatically adjusted. The resulting 16 bit images were analysed using the GenePix Pro 6.0 software. Data were processed using the MAnGO software [28]. The background level was calculated using morphological operators and subtracted. Raw data were normalized using the print-tip Loess method [29]. The array data were submitted to the NCBI Gene Expression Omnibus public depository, entry E-MEXP-3173 for experiments in the *Δhap4* background and E-MEXP-3131 for experiments in the *Δyap1* background.

Statistical comparisons were performed using multiple testing procedures to evaluate statistical significance for differentially expressed genes. On each slide, the normalized expression log-ratios were averaged on all replicates of each probe. A moderated t-test, e.g. a Student-like test [30], was computed. An error rate (p-value) is associated with each test value. Differentially expressed genes are selected with two criteria: p-value <0.005 and fold-change FC>|1.5|.

To reveal the changes in gene expression in the three different contexts (ScHap4, HpHap4-A, HpHap4-B), the heat map hierarchical clustering method of the heat map function of the R package “stats” was applied to the normalized log2 ratios (intensity of WT versus intensity of Mutant) obtained from the whole microarray experiments in the *ScΔhap4* genetic background and on the normalized median signals obtained for the different conditions in the *ScΔyap1* background.

Principal Component Analysis (PCA) was used to reveal unknown trends in the data and explore the correlations between mutants [31]. This method was applied to the same data (normalized log2 ratios) than those treated with the heat map clustering method.

Gene Ontology categorization was done with T-profiler tool. This tool uses the t-test to score changes in the average activity of pre-defined groups [32].

### Real-time RT-PCR experiments

Total RNA was extracted from *H. polymorpha* wild-type strain (NCYC495 *leu1-1*) and *Hp*Δ*Hap4-A* after growth on glucose and in 0.5 mM H_2_O_2_ for wild-type and *Hp*Δ*Hap4-B* in the same conditions as described previously. Three independent biological replicas were used. Reverse transcription experiments were performed with 5 µg of total RNA using reverse PCR primers as gene specific primers and superScriptIII as reverse transcriptase (Invitrogen, Carlsbad, CA). PCR primer pairs are listed in Table 2.

**Table 2:** List of *H. polymorpha* genes and corresponding primers used for qRT-PCR.

Hp gene name*	Oligomer name	Sequence 5'-3'
*HpFTR1*	FTR1For	TCCGGTCCAGGTACTTATAACATC
	FTR1 Rev	ATCAAAAGCAAGGTCACAATGACT
*HpYAP5*	YAP5For	TGGGAATCCGCAAAAGAGAGAAT
	YAP5Rev	AGAATTCGGGGATCTGAAAAGCA
*HpFRE4*	FRE4For	AGCCGAAGTCGATACTGACA
	FRE4Rev	TCTTGTACGAGCTGGTCGAT
*HpFRE3*	FRE3For	GACTCGGAGGAGCCAATCTG
	FRE3Rev	CCAAGCAAAGTTTCCCGCAA
*HpFRE2*	FRE2For	GAGCTGAAGTGGGTGGC
	FRE2Rev	GGCACAGGTCTGGCTTC
*HpCCC1*	CCC1For	CCTAGCTGCCCGTTCAGAAT
	CCC1Rev	ATCATCGTCTTGGGGTCAGC
*HpGPX1*	GPX1For	AAGGTCGACGTGAATGGTCCTAATG
	GPX1Rev	GATGTCTTCGGAAATCTTGGACGGA
*HpARG5,6*	ARG5-6For	AATTGCATCCGGCTCTACATCG
	ARG5-6Rev	CTGCTGTGAAGATGTTGTCGGT
*HpAAD1* **	AAD1For	TCTGTAGTGAAAGCTGGGTCG
	AAD1Rev	CTCGTTGACTGGGAAGTAGCA
*HpAAD2* **	AAD2For	GCTTCTTCGGATTGACCCAG
	AAD2Rev	AGTTTCAGCTTGATAGCGGC
*HpACT1*	ACT1For	CTCTGGTGACGGTGTTACCC
	ACT1 Rev	TGGTCGAAGTCAAGAGCCAC
*HpSDH1*	SDH1For	GGCTTGCCATTGGAGGATCT
	SDH1Rev	CCATGGTGATGGCTCTCGAA
*HpCYC1*	CYC1For	AGGTTCTGCTAAGAAGGGTGC
	CYC1Rev	CGGACATGGTCTGTTCGTTC
*HpMDH1*	MDH1For	CGAGGTGCTCAAGTCCAAGA
	MDH1Rev	AGAGCGTCGTAGGTCTCCTT
*HpCOX5-A*	COX5-AFor	AGAGCACTCGTCTTACGGGA
	COX5-ARev	TTCTCGTCTGGGGTGAGGTA

* The *H. polymorpha* gene names are given according to their homologues in *S. cerevisiae.*

**In the case of the Aryl-alcohol dehydrogenase genes (*AAD* genes, seven in Sc) we found only two genes in Hp and tested both. Priming with the AAD1 oligomers did not work and the experiment was not carried further.

cDNA templates were diluted to 1/500 for expression measurements. Standard conditions were used with the "Maxima SYBR Green qRT-PCR master mix" kit (Fermentas), in a Light Cycler System (Roche Diagnostics; DNA denaturation at 95°C for 10s, followed by 40 cycles of 60°C for 10s and 72°C for 15s). The specificity of each PCR reaction was checked by measuring fluorescent signals during melting curve analysis. Gene expression was calculated relative to the transcripts levels of the gene *HpACT1* which was shown in previous experiments to be constitutively expressed in our conditions. The statistical analysis was performed with the REST software (Qiagen).

## Results

### 1. Heterologous transcriptomic assays revealed that the two HpHap4 proteins behaved differently in the *ScΔhap4* genetic background

All previous experiments showed that both *H. polymorpha* Hap4 proteins can functionally replace the *S. cerevisiae* Hap4 protein; nevertheless, HpHap4-B was always less efficient than HpHap4-A for all criteria examined [12]. We also showed that *HpHAP4-B* has a role in iron homeostasis, reminiscent of what is observed for its orthologues in *Aspergillus nidulans* [21], *Candida albicans* [22] and *Schizosaccharomyces pombe* [19]. While both genes activate *CYC1* expression, we felt this was not enough to monitor their transactivation capacities. Therefore we compared the effects of overexpressing *HpHAP4-A*, *HpHAP4-B* and the native *ScHAP4* in *S. cerevisiae,* in transcriptomic assays. First, we examined genes which were up-regulated by *ScHAP4* overexpression (Tables S1, S2 and S3) and compared these data to previously reported assays with *ScHAP4* [5], [33]. As expected, most genes coding for the respiratory chain (Table S1) and enzymes of the TCA cycle (Table S2) were found to be under the control of *ScHAP4*, a result coherent with published data and the known function of this regulator. However, we detected a lesser number of genes encoding the mitochondrial translation apparatus than was previously described (Table S3) The reason for this discrepancy is unclear, but we noticed that if this functional class is represented in each experiment, the specific genes among this class are quite variable. Altogether, the overexpression of *ScHAP4* enhances, as expected, the expression of the many genes necessary to ensure the proper function of mitochondrial metabolism.

**Figure 3:**
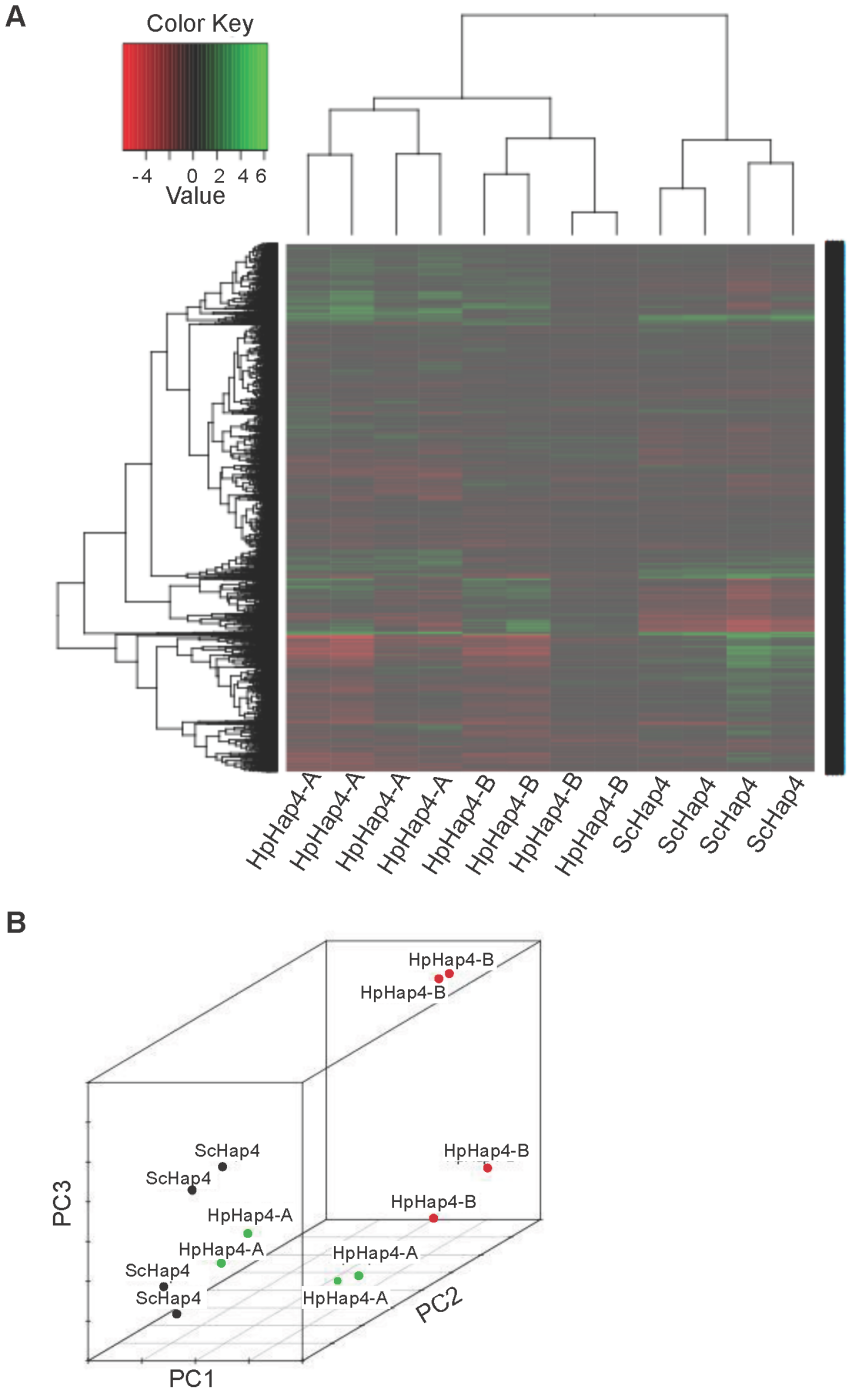
Analysis of transcriptomic data of the *Sc*Δ*hap4* strains. The analysed data are the normalized log2 ratios (intensity in WT strain versus intensity in the mutant strains) obtained from the microarray experiments (four biological replicates per condition) in the *ScΔhap4* genetic background. The WT strain is defined as *ScΔhap4* plus the empty plasmid pBFG1 and the three mutant strains as *ScΔhap4*plus pBFG1 containing the *ScHap4*, the *HpHAP4-A* or the *HpHAP4-B* genes. A: Heat map clustergram. This figure reveals the changes in gene expression between experiments. The experiments and the genes are clustered in a tree from their Pearson correlation coefficient values. Gene expression level is represented on a heat map by colour level (green when overexpressed and red when underexpressed compared to the WT strain). B: Presentation of the experiments in the 3D space of the principal components. The two axes of the figure are the three first principal components determined by Principal Component Analysis (PCA). They explain, respectively, 36%, 27% and 16% of the variance.

This being established, we examined global gene expression in conditions where either *HpHAP4-A* or *HpHAP4-B* was overexpressed (Fig 3A).

Fig 3B is a three dimensional “Principal Component Analysis” (PCA) of the different sets of data which represent in total about 80% of the variability (details for each principal component are given in the legend of the Figure). It clearly separates the *HpHAP4-B* data from the two groups representing *ScHAP4* and *HpHAP4-A* data. While the latter are not identical, they ought to have some common effects, exemplified when one examines detailed results for genes encoding the respiratory chain components (Table S1) and those for the TCA cycle (Table S2). (i) Most genes from these two lists are indeed regulated by *ScHAP4* and *HpHAP4-A* and one can conclude that in *S. cerevisiae*, the function of *HpHAP4-A* partially overlaps that of *ScHAP4*. (ii) In contrast, *HpHAP4-B* does not control this same set of genes (none were found significantly regulated by it). The genes differentially regulated by *HpHAP4-B* are listed Table 3. They are a limited set, involved in cell wall formation, dNTP synthesis or expressed in hypoxia/anaerobiosis. Interestingly, several of them were previously detected in our studies of *ScYAP1* [26].

**Table 3:** Genes up-regulated by *HpHAP4-B* in the Δ*hap4* background.

ARE*	Gene	Presumed function	r-factor**	p-value
y	*SEO1*	Putative permease; Sulfoxyde Ethionine resistance.	2.79	0.000
y	*RPL019B*	Ribosomal protein	2.06	0.006
y	*AAC3*	ATP/ADP translocase, anaerobically expressed	2.72	0.000
y	*COS111*	Detected in mitochondria	2.07	0.004
y	*PHO89*	Phosphate metabolism	2.25	0.003
n	*BSC1*	Similar to flocculin	3.23	0.000
y	*YDL038C*	Merged with YDL039C, a pheromone regulated protein	5.85	0.000
y	*PRM7*	Pheromone regulated protein; response to drug	3.46	0.000
y	*HO*	Required for gene conversion of MAT	3.26	0.000
y	*HXT15*	Hexose transporter, induced in low level of glucose	2.06	0.000
y	*SOR2*	Fructose or mannose metabolism?	2.01	0.000
y	*ARO10*	Phenyl pyruvate decarboxylase, first step of the Ehrlich pathway	4.43	0.001
y	*YEL057C*	Telomere maintenance?	2.31	0.002
y	*AGX1*	Alanine:glyoxylate aminotransferase. Glycine synthesis in gly/eth.	2.01	0.000
y	*ALG13*	Glycosyltransferase, ER	2.17	0.005
y	*FMP48*	Found in mitochondrial proteome	2.01	0.000
y	*BIO2*	Biotin synthesis	2.25	0.000
y	*PEX18*	Required for peroxisome targeting	2.15	0.002
y	*TIR3*	Cell wall mannoprotein/required for anaerobiosis	2.25	0.000
y	*RNR2*	dNTP synthesis	2.05	0.002
n	*INO1*	Inositol phosphate synthesis	6.84	0.003
y	*FAR1*	Cell cycle arrest	2.31	0.000
n	*ANB1*	EIF-5A, anaerobiosis gene	4.67	0.000
y	*SFC1*	Succinate-fumarate transporter	2.74	0.000
y	*HXT16*	Hexose transporter, repressed in high glucose	2.16	0.000
y	*PTR2*	Peptide transporter	4.02	0.004
y	*RPL38*	Ribosomal protein	2.07	0.002
y	*YLR413W*	Unknown function	3.82	0.000
y	*RPS1A*	Ribosomal protein	2.08	0.004
y	*HXT2*	Glucose transporter, induced at low levels of glucose	3.49	0.000
n	*FET3*	multicopper oxidase; required for high affinity iron uptake	2.04	0.000
y	*HAS1*	Helicase, rRNA processing	2.30	0.000
n	*RAS1*	Ras protein signal transduction	2.16	0.000
y	*YOR121C*	Dubious, overlaps YOR120W	2.09	0.003
y	*ALD6*	Converts acetaldehyde to acetate. Binds mit OM in oxidative stress	2.11	0.004
y	*ODC1*	Mitochondrial transporter, involved in lysine and glutamine biosynthesis.	2.04	0.007
n	*GUP2*	Proton symport of glycerol	2.09	0.000
y	*NIP7*	Nucleolar protein required for 60S ribosome subunit biosynthesis	2.14	0.000

* indicates the presence(y)/absence (n) of a putative Yap1 binding sites in the promoter as obtained from YEASTRACT (http://www.yeastract.com/index.php) database.

** r-factor is the ratio between the normalized value in the strain *Δhap4* carrying the empty BFG1 plasmid and the value obtained in the strain *Δhap4* carrying BFG1 plus *HpHAP4-B*.

### 2. *HpHAP4-B* is able to replace *YAP1* in *S. cerevisiae*

**Figure 4:**
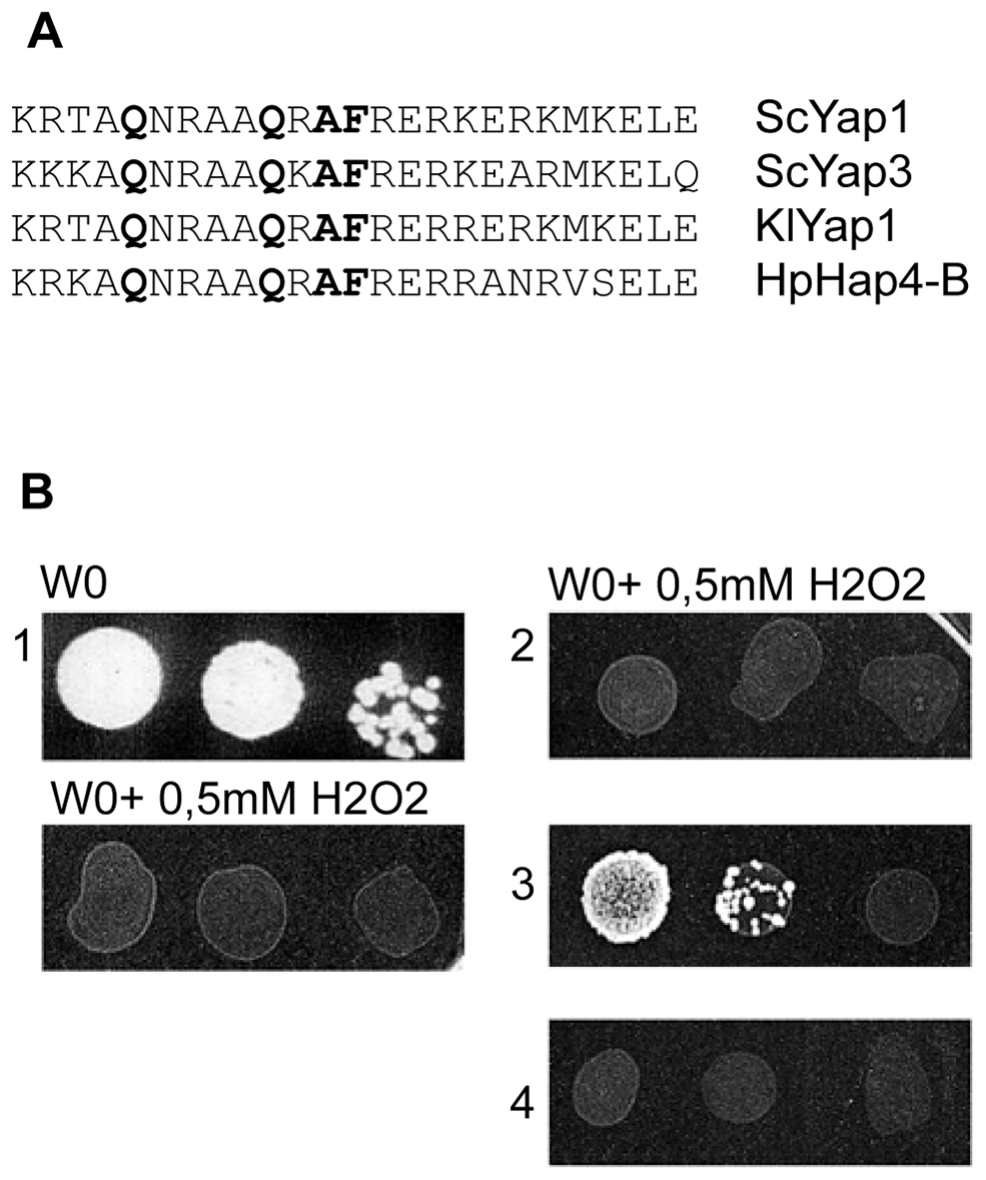
Functional comparison between *ScYAP1* and the *HpHAP4-B* genes. Part A: Comparison of the new bZIP type motif identified in HpHap4-B with the YAP family motif. Q234, Q239, A241 and F/Y242 are four basic regions (DNA-binding sites) characteristic residues of the YAP protein family which are rarely or never observed in other bZIP proteins [34]. Part B: Heterologous complementation of the growth deficiency of *S. cerevisiae* Δ*yap1* in the presence of H_2_0_2_ by the *HpHAP4-B* gene. The gene is expressed on a multicopy plasmid (pBFG1, see *Methods*) and the growth monitored on minimal medium (W0) containing 0.5 mM of H_2_0_2_. 1: *S. cerevisiae* Δ*yap1* strain (with or without H_2_0_2_). 2:Δ*yap1* strain with empty plasmid BFG1. 3: Δ*yap1* with BFG1 plasmid containing *HpHap4-B*. 4: Δ*yap1* with BFG1 plasmid containing *HpHap4-B* with deleted bZIP domain (see Methods). Tests 2, 3 and 4 were performed on medium containing H_2_0_2_. Strains were grown on minimal glucose medium supplemented with the necessary aminoacids at 28°C for 5 days.

The additional bZIP-type DNA-binding motif observed in many proteins encoded by putative *HAP4* orthologues, especially when the species harbouring them are phylogenetically distant from *S. cerevisiae*, is a well conserved motif of 25 amino acids which is similar to the DNA-binding motif of the *S. cerevisiae* YAP family of proteins (Fig 4A). It contains four characteristic amino acid residues, Q234, Q239, A241 and F/Y242 specific of the Yap family [34]. This sequence, called the "Basic Region" (abbreviated here as BR), is necessary for the ScYap1 transactivator to recognize its specific cis-DNA binding sequences (called ARE). The ZIP motif (leucine zipper), which is adjacent to the BR region, was not identified in these *HAP4* orthologues; only putative coiled-coiled motifs quite distant to the basic motif could be detected [12].

This result, as well as the data obtained from transcriptomic studies, prompted us to check if the presence of the BR motif provided the HpHap4-B protein with the capacity to act as ScYap1 does, and to functionally replace it in the *S. cerevisiae* host. As can be seen in Fig 4B, *HpHAP4-B* could correct the H_2_O_2_ hypersensibility of the *ScΔYap1* mutant as observed for the control (Sc*YAP1*). This response is specific for the *HpHAP4-B* gene; neither *HpHAP4-A* nor *ScHAP4* have this property (data not shown). Note that deletion of the sequence coding for the BR motif in the *HpHAP4-B* sequence abolished the above-mentioned property (Fig 4B), demonstrating that this capacity is mediated by it.

**Figure 5:**
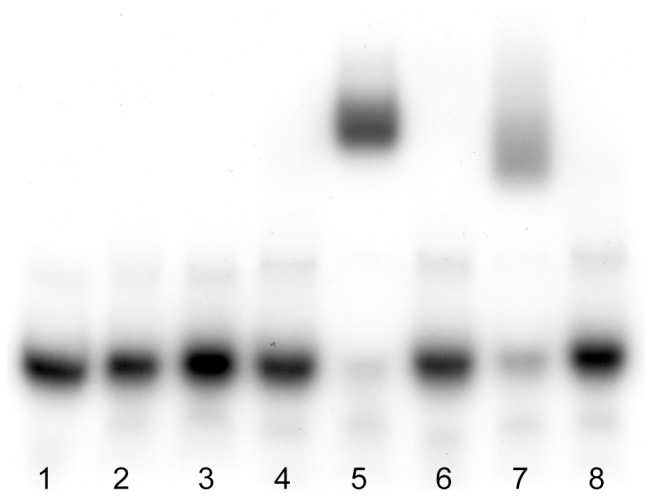
Gel shift experiment with a synthetic ARE sequence. Probe alone (lane 1), *S. cerevisiae* Δ*yap1* strain (lane 2), *Sc*Δ*yap1* with empty plasmid pBFG1 (lane 3), *Sc*Δ*yap1* with pBFG1 plasmid carrying *ScHAP4* (lane 4), pBFG1-*ScYAP1*(lane 5), pBFG1-*HpHAP4-A* (lane 6), pBFG1-*HpHAP4-B* (lane 7) pBFG1-*HpHAP4-B-bZIP* (devoid of Hphap4-B BR region; lane 8).

These data indicate that HpHap4-B is able to activate the expression of (at least some) ScYap1 target genes and to bind to the ARE sequence. To verify this hypothesis, we undertook a gel shift analysis as reported in Fig 5. The HpHap4-B protein slows the migration of the specific, labelled, ARE oligonucleotide on the gel (lane 7) as does ScYap1 (lane 5). This shift was absent in the negative controls (lanes 1–4 and 6), as well as when the BR motif was deleted from the *HpHAP4-B* sequence (lane 8), which confirms that DNA binding is mediated via the BR sequence.

### 3. How does *HpHAP4-B* correct the defective *ScΔyap1* phenotype: global gene expression analysis in the *ScΔyap1* context

**Figure 6:**
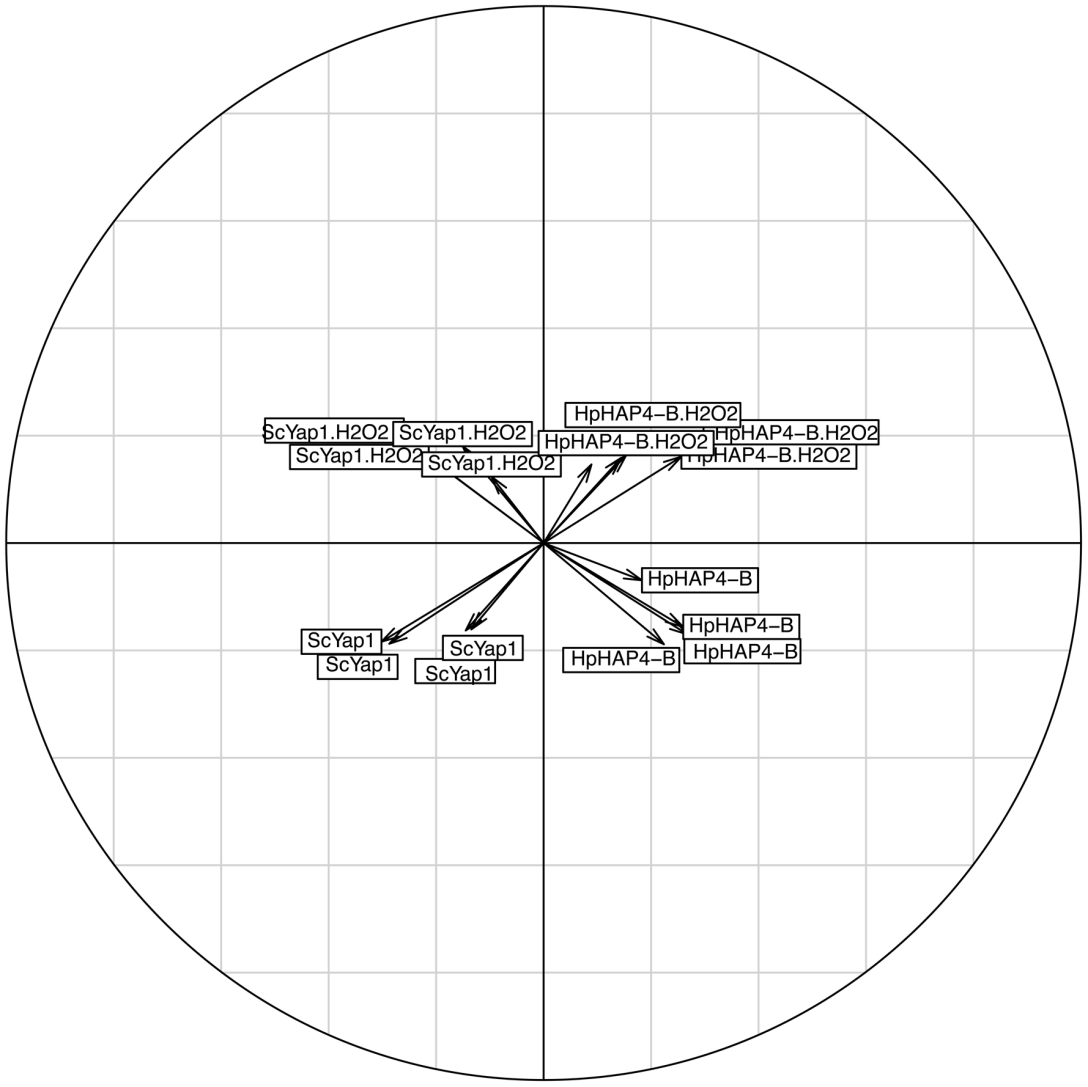
Principal Component Analysis of transcriptome data of the *ScΔyap1* strains. The analysed data are the normalized median signals obtained in two mutants and in two different growth conditions (with or without H_2_O_2_), compared to the WT by microarray experiments in a *ScΔyap1* genetic background. The WT is defined as *ScΔyap1* plus the empty plasmid pBFG1, and the mutants as *ScΔyap1* plus pBFG1 carrying either the *ScYAP1* or the *HpHAP4-B* gene. For each condition, four independent experiments were performed. The two axes of the figure are the second and the third first principal components and explain 7.2% of the variance.

We performed transcriptomic analysis of the effect of *HpHAP4-B* overexpression in the *ScΔyap1* context in the absence and presence of H_2_O_2_ and compared the data with the effect of *ScYAP*1 overexpression in the same conditions. The three first principal components of the PCA analysis performed on the complete set of transcriptomic data explain 94.7% of the variance of the data. The second and the third components explain 7.2% of the variance and separate Sc*YAP1* action from *HpHAP4-B* action on one side and the presence and absence of oxidative stress conditions (H_2_O_2_) on the other (Fig 6). Since both transactivators restore growth in the presence of H_2_O_2_, this strongly suggests that they achieve this endpoint acting upon some common target genes or functions (see below).

Target genes regulated by ScYap1 or HpHap4-B were classified according to their gene ontology (GO) functions (Table 4) and one can immediately note striking differences. If ScYap1 is, as expected, mostly geared towards ROS scavengers and chaperones, HpHap4-B is much more biased towards carbon assimilation. Such rapid analysis tells us that while both of them can cope with oxidative stress, their function is clearly different. In addition, a large number of genes can be regulated by both of them (a detailed list is provided in Table S4). For example, if one examines ROS scavenger, the following genes (*GSH1*, *GLR1*, *GTT2*, *GTO3* or *ECM4*, all involved in glutathione metabolism; *TRX2*, *TRR1* or *TSA2*, involved in thioredoxin metabolism; the superoxide dismutases *SOD1* and *SOD2* and their chaperone *CCS1*; *CUP1-A* and *B* and *CTA1* encoding the catalase A) are up-regulated by Sc*YAP1* specifically in the presence of H_2_O_2_, confirming previous observations [26]. Some of these genes are also regulated by both *ScYAP1* and *HpHAP4-B* (such as *GTT2* encoding gluthatione transferase or *TSA2* encoding thioredoxin peroxidase) and therefore belong to this common core of targets while others such as *CTT1*, which encodes the cytoplasmic catalase, or *GRX1* (the glutaredoxin which participates to glutathione metabolism) are regulated only by *HpHAP4-B*. However, the combination of each transactivator target genes is large and varied enough to achieve the response to oxidative stress, even though the sets of genes do not completely overlap.

**Table 4:** Gene ontology categories of the genes regulated by HpHap4-B and ScYap1.

*HpHAP4-B* overexpression in presence of H_2_O_2_				
Gene ontology category	t-value	E-value	Mean fold change	Number of ORFs
Fructose transporter activity	9,49	<1.0e–15	4.969	13
Carbohydrate metabolism	8,01	1.54e–12	1.221	153
Response to stress	5,76	1.17e–5	0.582	283
Protein catabolism	4,45	1.19e–2	0.722	120
Oxidoreductase activity, acting on peroxide as acceptor	4,39	1.56e–2	2.572	10
ER to Golgi transport	−4.85	1.71e–3	−1.064	51
Transporter activity	−4.91	1.26e–3	−0.409	330
Biosynthesis	−5.14	3.82e–4	−0.291	676
Nucleotidyltransferase activity	−5.21	2.62e–4	−1.119	59
Protein biosynthesis	−11.58	<1.0e–15	−0.887	366
Ribosome biogenesis and assembly	−12.22	<1.0e–15	−1.485	185
***ScYAP1*** ** overexpression in presence of H_2_O_2_**				
Aldehyde metabolism	7,44	1,47E–11	6,93	18
Oxidoreductase activity, acting on the CH-OH group of donors, NAD or NADP as acceptor	5,81	9,12E–07	3,034	54
Oxidoreductase activity, acting on CH-OH group of donors	5,29	1,79E–05	2,584	62
Oxidoreductase activity	5,14	4,01E–05	1,348	213
Membrane	−3,61	4,37E–02	−0,378	767
Intracellular	−3,69	3,22E–02	−0,047	3483
Large ribosomal subunit	−4,02	8,46E–03	−1,337	113
Protein metabolism	−4,3	2,49E–03	−0,401	936
Biosynthesis	−4,3	2,49E–03	−0,487	711
Cytosolic ribosome (sensu Eukarya)	−4,33	2,17E–03	−1,28	141
Structural constituent of ribosome	−5,15	3,80E–05	−1,294	192
Ribosome	−5,21	2,76E–05	−1,195	226
Structural molecule activity	−5,29	1,79E–05	−1,075	281
Protein biosynthesis	−5,44	7,78E–06	−0,93	379
Macromolecule biosynthesis	−5,67	2,08E–06	−0,821	503

The same holds true for genes encoding heat shock proteins (*MDJ1*, *HSP26*, *SSE2, HSP42*, *HSP78*, *HSP31*, *HSP104* and others). Globally, stress-induced genes represent 40 to 50% of genes regulated by *YAP1* or *YAP1* and *HpHAP4-B*, but only 12% of those genes were regulated only by *HpHAP4-B.*

We previously reported [26] that *ScYAP1* also controls several genes involved in carbon metabolism, which we hypothesized to be necessary for redox balance (several genes of the pentose phosphate pathway were up-regulated in these conditions). When *YAP1* is overexpressed, only some of these genes are identified (such as *GND2* which catalyses a NADPH regenerating reaction in the pentose phosphate pathway) but we found other genes that should ensure a correct redox balance (several dehydrogenases in particular). Surprisingly, we found many genes involved in carbon metabolism among the *HpHAP4-B* specifically up-regulated genes (glycolytic genes such as *GPM2*, *MRK1*, *TLK2*, and several coding for enzymes of the carbohydrate storage pathway such as *GLK1*, *TPS1*, *NTH1* and *TPS2*).

Finally, genes involved in protein degradation, inositol metabolism, intracellular trafficking and cell wall were found up-regulated both in conditions of the presence and absence of oxidative stress, a situation previously described [26]. Unexpectedly, we also found that a great fraction of the genes which were regulated in common by both transactivators are involved in translation and the majority of them participate in cytoplasmic ribosome biogenesis (about 90% of the genes belonging to the translation functional categories). This amount was much higher than expected as their normal contribution to the functional categories but might be artefactual, reflecting only the effect of overexpression.

The lists of up-regulated genes, which are under control of either *ScYAP1* or *HpHAP4-B* or both in H_2_O_2_ stress condition, are presented in Table S4.

### 4. The two HpHap4 proteins have different functions in their natural host *Hansenula polymorpha*

All preceding experiments have been performed in a heterologous system, where the *H. polymorpha* genes were analysed in *S. cerevisiae*. They all point to the idea that *HpHAP4-A* seems to be the functional orthologue of the *S. cerevisiae HAP4* gene (it regulates mostly the same targets and fully complements *ScHAP4* function). Conversely, *HpHAP4-B* is much less efficient at replacing *ScHAP4*, can bind the ARE *cis*-binding site of the *ScYAP1* gene and replace it, even though less efficiently, *in vivo.* Altogether these data indicates that *HpHAP4-B* should be able to regulate (at least some) *ScYAP1* targets. These apparent functional differences remained to be examined in the natural host *H. polymorpha*.

**Figure 7:**
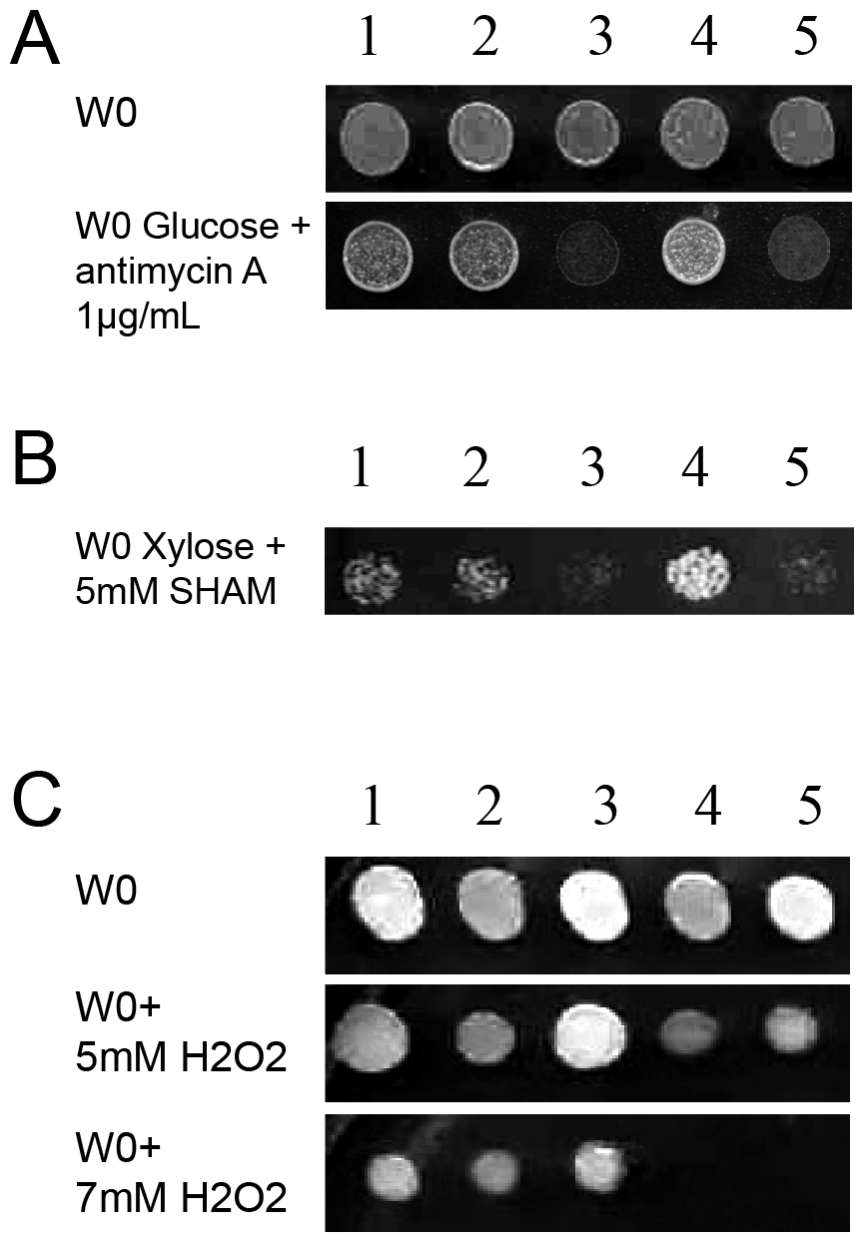
Growth of different *H. polymorpha* strains on media containing either antimycin A, SHAM or hydrogen peroxide. A: *H. polymorpha Hp*Δ*hap4-A* and double knock-out strains, but not *Hp*Δ*hap4-B* deletion mutant, are sensitive to antimycin A on glucose. B: Growth on xylose plus SHAM or H_2_0_2_. 1: *Hp*NCYC495*leu1_1* strain. 2: *Hp*NCYC495*leu1_1* with pYT1 (empty plasmid, see Material and Methods). 3: *Hp*Δ*hap4-A*. 4: *Hp*Δ*hap4-B*. 5: *Hp*Δ*hap4-A Hp*Δ*hap4-B* (double knock-out strain).

The two genes were deleted, individually or in combination (double mutant), in *H. polymorpha* and the resulting phenotypes were analysed. Fig 7 shows the growth of the different mutant strains in presence of various drugs. Growth of strains carrying the single deletion of *HpHAP4-A*, or the same deletion associated with the *HpHAP4-B* deletion, are impaired in the presence of 1 µg/ml of antimycin A. This drug is a well-known inhibitor of electron transfer from quinone to cytochrome b in mitochondria [35]. The controls (wild-type strain with or without the empty plasmid) as well as the single deletant *HpΔhap4-B*, grow normally (Fig 7A). Moreover, the same mutants were also sensitive to salicylhydroxamic acid (SHAM) during growth on non-fermentable sugar xylose (Fig 7B). SHAM is a potential inhibitor of fungal alternative oxidases [36], the branch of mitochondrial electron transport chains that transfers electrons from the ubiquinol pool directly to molecular oxygen. AOX (alternative oxidases) were reported for many fungi (reviewed in [37]). However, to our knowledge they had not yet been described for *H. polymorpha*.

*HpΔhap4-B* mutant and the double disruption mutant were sensitive to H_2_O_2_, while the *HpΔhap4-A* strain grows normally (Fig 7C). These results clearly indicate different functions for the two *HpHAP4* paralogues, one related to carbon and energy metabolism and the other involved in oxidative stress.

In order to go beyond the observed phenotype, we examined by qRT-PCR the expression of some genes that could be the targets of these two transactivators in *H. polymorpha*. Choosing these genes is not an easy task since even if the transactivator function is conserved, it may not be achieved through exactly the same set of target genes. We based our choice on a few genes regulated in the heterologous experiment (see Table S4 for *YAP1* and *HpHAP4-B*) and searched their orthologues. For *HpHAP4-A*, we selected a few genes encoding components of the respiratory chain or Krebs cycle (*HpSDH1, HpMDH1, HpCYTC1, HpCOX5A*) and for *HpHAP4-B*, we selected the targets based on their function in redox control. We examined the expression of several *H. polymorpha* genes possibly involved in iron homeostasis (*HpFRE2, HpFRE3, HpFRE4, HpFTR1, HpCCC1*), one gene encoding an aryl-alcohol dehydrogenase (*HpAAD2*), the gene encoding glutathion peroxidase (*HpGPX1*) and another encoding one step of the arginine biosynthesis pathway (*HpARG5,6*). The orthologue of the *YAP5* gene, a transcriptional regulator which regulates vacuolar iron storage in *S. cerevisiae* has also been included. Other genes (such as heat shock proteins) would have been worthy of testing but they were too many copies of the different genes to choose soundly among them. Results are presented in Table 5.

**Table 5:** qRT-PCR of *HpHAP4-A* and *HpHAP4-B* regulation of gene expression in *H. polymorpha.*

Part A: regulation by HpHAP4-A in glucose		
Gene	Expression ratio Δhap4-A/WT	P(H1)
*ACT1*	1.000	-
***SDH1***	**0.502**	**0.032**
***CYTC1***	**0.610**	**0.000**
***MDH1***	**0.596**	**0.017**
*COX5*	0.629	0.097

Part B: regulation by HpHAP4-B in glucose		
Gene	Expression ratio Δhap4-B/WT	P(H1)
ACT1	1.000	-
FTR1	0.595	0.203
**YAP5**	**<0.001**	**0.000**
**FRE4**	**8.634**	**0.013**
CCC1	0.580	0.160
AAD2	1.072	0.751
FRE3	1.120	0.648
FRE2	0.474	0.191
GPX1	0.491	0.095
ARG5,6	0.572	0.095

Part C: regulation by Hp HAP4-B in glucose plus H_2_0_2_		
Gene	Expression ratio Δhap4-B/WT	P(H1)
ACT1	1.000	-
**FTR1**	**0.018**	**0.036**
**YAP5**	**0.05**	**0.000**
**FRE4**	**0.366**	**0.000**
**CCC1**	**5.063**	**0.037**
AAD2*	2.06	0.365
FRE3	1.181	0.602
FRE2	1.159	0.747
GPX1	0.699	0.508
ARG5, 6	0.636	0.506

The P(H1) value indicates the probability that the difference between the sample and control groups is due only by chance and was analysed with the REST software (Qiagen, see Material and Methods for more details). Statistically significant results are shown in bold. The case of AAD2* is not conclusive since the data were obtained in two different experiments (two qRT-PCRs, regulated and reproducible and a third one, performed later, which was not).

We assayed *HpHAP4-A* in glucose which is a fermentescible substrate. If a regulation does exist, we expect the ratio to be around 2, based on our experience with *S. cerevisiae*. We used galactose in the latter case because it is a fermentescible substrate with no repression of the respiratory function (as does glucose) and we observed regulatory ratio to be around 2 [5]. In *H. polymorpha*, there is no glucose repression so that the experiment mimics, as far as carbon source is concerned, galactose in *S. cerevisiae*. We can therefore satisfactorily conclude that the genes *HpSDH1*, *HpCYTC1*and *HpMDH1* are regulated by *HpHAP4-A*. The fourth one (*HpCOX5*) is statistically at the limit and may well be regulated.

As for *HpHAP4-B*, we observed in presence of oxidative stress transcriptional control of *HpFTR1, HpYAP5, HpFRE4 and HpCCR1*. The case of *HpAAD2* is unclear (see legend). It is also interesting to note that *HpYAP5* and *HpFRE4* are under control of *HpHAP4-B* even without any oxidative stress, but for the latter the regulation is opposite.

Although the number of genes which were analysed is limited, these results are compatible with our hypothesis, all the more since *HpHAP4-A* is induced by respiratory substrates (data not shown) and *HpHAP4-B* is regulated by iron [12].

### 5. Distribution of the two motifs (N-Hap4 and BR) in proteins of fungal species

**Figure 8:**
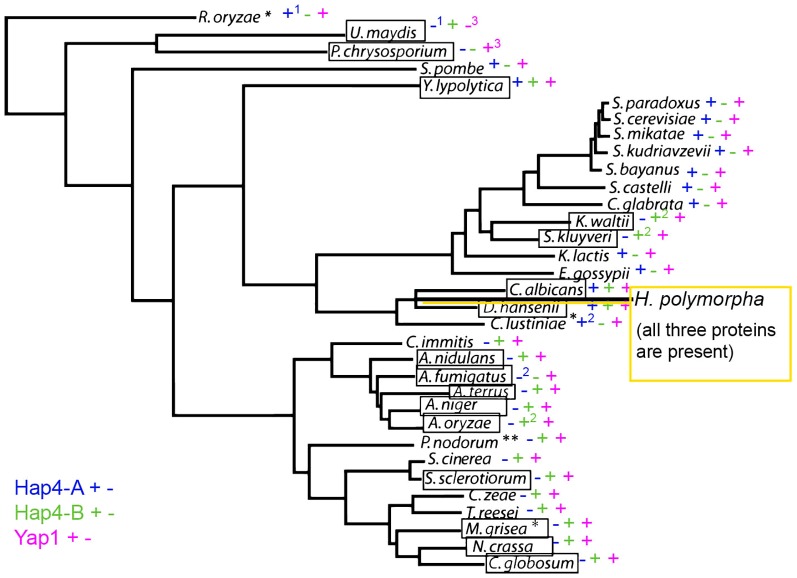
Phylogenetic tree of fungi in relation with the presence/absence of HpHap4-B-type of proteins. The phylogenetic tree was adapted from [49]. Species in which proteins such as HpHap4-B (containing both the N- Hap4 and the BR motifs) could be identified are boxed. Aligned with each species, the presence of proteins of the Yap1 type (Yap1 BR motif), HpHap4-B type (BR plus N-ter motifs) and HAP4-A type (N-ter motif) of protein is indicated next to each species. The position of *H. polymorpha*, which is not on the tree, is indicated, underlining that this yeast belongs to the clade where *C. albicans* and *D. hansenii* are localized. * These species were not correctly annotated; for *P. nodorum*, the BR and N-ter motif are overlapping and degenerate while for *U. maydis*, the protein annotated as Yap1 turned out to contain also an N-ter motif and belongs therefore to the HpHap4-B family of proteins.

The three types of proteins, Yap1, HpHap4-A and HpHap4-B share specific motifs as indicated in Fig 1: the N-ter motif present in HpHap4-A and HpHAP4-B, the Cysteine Rich domain (or CRD) present in HpHAP4-B and Yap1, and the BR motif present in HpHAP4-B and Yap1. The simultaneous presence of the N-ter and BR motif has already been examined in Ascomycetes, and shown to be associated with Hemiascomytes phylogenetically distant to *S. cerevisiae* and Euascomycetes for which the genome sequences were available [12]. The N-ter motif of ScHap4 and HpHap4-A is not found in eukaryotes in general, but seems to be fungi-specific. We placed the presence/absence of the Hap4 N-ter and Yap1 BR motifs into the phylogenetic tree and examined the relations between them in fungi for which we disposed of complete genome sequences (Fig 8). When proteins contained both the N-ter and the BR motif, they were similar to Hap4-B and noted as such. The Yap1 type of protein is present in all species. However, the *U. maydis* Yap1, isolated and characterized as such [38] also has a N-ter motif just upstream the BR motif; this characterizes the protein as belonging to the HpHap4-B rather than to the Yap1 family. The HpHap4 type of protein (containing only the N-ter motif) is present in all hemiascomyceteous yeasts as well as *S. pombe* but absent in all Pezyzomycotina species.

Finally, the HpHap4-B structure containing both the N-ter and the BR motifs is absent in the *Saccharomyces* group and present in yeasts which are phylogenetically more distant such as *H. polymorpha*, *C. albicans* or *Y. lipolytica*, but surprisingly also in the clade of *K. waltii* and *S. kluyverii*. It was also present in all Pezyzomycotina species. Interestingly it was also present in the Basidiomyces *U. maydis*, a species more basal in the fungal tree than the others.

## Discussion

We present here comparative functional studies of the two Hap4-like proteins of the yeast *Hansenula polymorpha* performed in *S. cerevisiae*. The *H. polymorpha* genome sequence has only recently become publicly available. Using *S. cerevisiae* provided important insights and was probably essential to understand these new features of the Hap4 family of proteins: (i) HpHap4-A and HpHap4-B do not have redundant functions. (ii) HpHap4-A is a functional homologue of ScHap4 since it is essential for respiration and oxidative phosphorylation. The list of target genes, in large part, overlaps the target genes regulated by ScHap4, in particular the genes encoding the components of the respiratory chain. (iii) HpHap4-B bears some relation with ScYap1, which role is to cope with oxidative stress. We deleted the two genes in their natural host, *H. polymorpha*, and examined the phenotype of the relevant mutants. The results are fully coherent with what was deduced from the heterologous experiment: in the case of *HpHAP4-A*, presumed to be similar to *ScHAP4*, the deletion mutant was indeed hypersensitive to antimycin A and SHAM, inhibitors of different components of the respiratory chain. This was not the case for *HpHAP4-B* which, when deleted, led to a phenotype of hypersensitivity to oxidative stress caused by H_2_O_2_, in good agreement with its ability to functionally replace Sc*YAP1*. This hypothesis was reinforced by the qRT-PCR analysis.

### 

#### What is the mechanism used by HpHAP4-B to regulate gene expression?

Why these differences were observed in *S. cerevisiae* is surprising. We have previously shown that HpHap4-B can bind the core proteins of the HAP complex, though to a lesser extent than HpHap4-A [12]. Global gene expression studies reveal that its action partially overlaps ScYap1, and all the more so in a *Δyap1* context. This indicates that it probably can bind *in vivo* to the promoter of some genes that are ScYap1 gene targets (via the ARE sequence), extending what we observed *in vitro*. The mechanism of action of the protein is not yet known. An examination of the cis-binding sites present upstream of the genes regulated by either *ScYAP1* only, or *HpHAP4-B* only or both *ScYAP1* and *HpHAP4-B*, using the YEASTRACT (http://www.yeastract.com/) documented targets, does not show any major differences. Most of the genes have an ARE binding site in their promoter, but conversely, not all genes regulated by *YAP1* are regulated by *HpHAP4-B*. This may reflect that, *in S. cerevisiae*, some target sites could accept a Yap1 homodimer while some others absolutely require a heterodimer to be made with another Yap protein, an association which HpHap4-B is probably not capable of since it does not contain the "ZIP" part of the bZIP motif [12]; the situation in *H. polymorpha* may be different and one cannot exclude that promoters of genes regulated by HpHap4-B contain the two motifs (CCAAT and ARE) in close association.

The structure of the protein should also be considered. Conformational changes (the N-Hap4 and BR motifs are next to one another) could lead to the masking of one motif in some conditions. Our results cannot provide definitive answers on the respective role of these two proteins nor on the molecular mechanisms that control them, but may open the way to elaborate plausible hypotheses that should be tested directly in *H. polymorpha*.

#### The distribution of the HpHap4-B complex motif suggests a common origin for the Hap4 and Yap1 families of proteins

We have identified the N-Hap4 motif (16 aas), in all ascomycetes and (even though it is less conserved) in some Basidiomycetes. In several species, especially those phylogenetically distant from *S. cerevisiae*, it is associated with the BR motif of a Yap1p-like bZIP motif. The question of how this new association (N-ter plus BR) is distributed is therefore of interest. From the survey in Fig 8, it is clear that the presence of the HpHap4-B type of protein is scattered among the different clades. For example, outside of the Hemiascomycetes group, it is absent in *S. pombe* and *S. japonicus* but present in the Basidyomycetes *U. maydis*. It is present within one clade containing *K. waltii* and *S. kluyverii* and present in all the Pezyzomycotina species we examined, but the motif sequence was found degenerated in *P. nodorum*. The simplest way to explain these variations is to postulate that the fungal ancestor protein had the three motifs (N-ter, BR and CRD) which have been independently lost in different clades through evolution and speciation; degenerate motifs being witnesses of this evolution. Such a mechanism could have been used for neo- or sub-functionalization of these proteins.

For example, the HpHap4-B type of protein was not detected in *S. cerevisiae* and related species where the many duplications of the Yap genes family allowed partition of the function. While HpHap4-B is involved in oxidative stress, iron metabolism and storage and carbohydrate control, in *S. cerevisiae*, Yap1 is more specialized in oxidative stress, Yap5 is an iron responsive transcriptional activator [39] and Hap4 is specialized in the control of carbohydrates.

**Figure 9:**
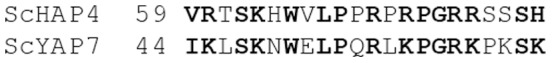
Identification of a “Relic sequence” in *S. cerevisiae*. Upper part (Hap4) is the N-ter Hap4 motif with small extensions (the motif itself spans aminoacids 60 to 76). Bottom part (Yap7) is a small sequence in the Yap7 protein which is 68% similar to the upper sequence. Yap7 is a member of the bZIP family of *S. cerevisiae* transactivators. Its function is not precisely known [50]. Refer to Fig 1 for the various motifs of these proteins.

If this evolutionary scenario is true, one should find, in the different yeast species, proteins with various combinations of the N-Hap4, BR and CRD domains (the cysteine-rich CRD domain is also part of the Yap1 sequence and critical for Yap1-mediated resistance to oxidative stress [40], [41] and can be found in the HpHap4-B type of proteins). It should be also possible to detect “relics” of these specific motifs in some species. It is therefore noteworthy that a close examination of *the S. cerevisiae* Yap proteins revealed a degenerate N-Hap4-B motif in the Yap7 protein (68% similarity, Fig 9). In addition, the fact that in *U. maydis*, which is less divergent relative to the putative ancestral fungi than the Hemiascomceteous yeasts, only one protein able to respond to oxidative stress has been found and that it has a Hap4-B type of protein (i.e. containing the three motifs N-ter, BR and CRD) reinforces this scenario [38].

In mammals, the fact that bZIP proteins can bind CCAAT sequences (cited in [42]) and that the bZIP ancestor is supposed to have functioned as a homodimer [43], [42] fits very well with the presence of a common ancestor for *HAP4* and *YAP1*. It is interesting to note that if we compare the ScHap4 and SpPhp4, proteins which are encoded by the phylogenetically distant yeast species *S. cerevisiae* and *S. pombe*, both regulate the mitochondrial components involved in respiration and both do so through the CCAAT-binding sequence. However their action is opposite: ScHap4 activates respiration in response to glucose derepression whereas SpPhp4 represses it in response to iron deficit [19]. This is one of the increasing examples of the complexity of regulatory network evolution where orthologues of target genes and transcriptional regulators are conserved but the regulatory strategy is changed in order to adapt to a different environment (reviewed in [44]).

#### Relation between carbohydrate metabolism, oxidative status and iron homeostasis

Data obtained in the course of this work, and from the literature, point to an intricate relation between the control of iron homeostasis, oxygen tension and carbon source. Recently, it was demonstrated that the CCAAT-binding factor of *Aspergillus nidulans* (composed of HapB, HapC and HapE, equivalent to Hap2, Hap3 and Hap5 in *S. cerevisiae*), senses the redox status of the cell [45], independently of the activator moiety HapX (the equivalent of HpHAP4-B). This relation is not surprising since the presence of excess iron during oxidative stress is deleterious to the cell, allowing the Fenton reaction to take place. Conversely iron is needed for many pathways, among which is the synthesis of the respiratory components. Iron homeostasis control is therefore also linked to the type of carbon sources, respiratory or fermentative, that are available. Carbon source utilization can be different from one species to another and has been studied in detail in the *Saccharomyces* complex by [46]. These species show a greater variability in terms of the ability to accumulate ethanol in the presence of oxygen and a gradual independence from oxygen in the pre-genome duplication species. In *Kluyveromyces lactis*, we recently showed that the KlHap1 protein, a transactivator involved in regulation of gene expression in response to heme and oxygen in *S. cerevisiae*, controls glucose transport [47]. The tight control of these three pathways was probably more primitive in the yeast progenitor but absolutely necessary, and a regulator of the *HpHAP4-B* type was probably the simplest way to coordinate all these actions. During evolution, more specialized functions appeared, explaining the highly redundant and specialized functions of the many regulators involved in iron control, oxidative stress control and carbon source assimilation in *S. cerevisiae.* Therefore and according to the environmental niche, proteins of some species lost one or more of the three motifs (N-Hap4, BR and CRD) found in the original protein.

The possibility to test in the future the global regulatory role of the *Hap4-B* type of proteins in different species will allow us to assess and refine these hypothesis.

## Supporting Information

Table S1 **Comparison of the regulatory ratios (WT versus mutant) obtained by overexpression of** ***ScHAP4 or HpHAP4A in ScΔhap4 mutant:*** **genes encoding respiratory chain components.**
(DOCX)

Table S2 **Comparison of the regulatory ratios (WT versus mutant) obtained by overexpression of** ***ScHAP4 or HpHAP4A in ScΔhap4 mutant:*** **genes encoding TCA cycle and related pathways enzymes.**
(DOCX)

Table S3 **Comparison of the regulatory ratios (WT versus mutant) obtained by overexpression of** ***ScHAP4 or HpHAP4A in ScΔhap4 mutant:*** **genes encoding components of the mitochondrial translation apparatus.**
(DOCX)

Table S4 **List of** ***S. cerevisiae*** **genes upregulated in presence of H202 when** ***ScYAP1*** **or** ***HpHAP4-B*** **were overexpressed in** ***ScΔyap1*** **mutant.**
(DOCX)
