# Supplementary material for: Functional Study of the Hap4-Like Genes Suggests That the Key Regulators of Carbon Metabolism HAP4 and Oxidative Stress Response YAP1 in Yeast Diverged from a Common Ancestor
Source: PLoS One. 2014 Dec 5;9(12):e112263. doi: 10.1371/journal.pone.0112263 (PMC4257542; doi:10.1371/journal.pone.0112263)
Supplement: Table S1 — Comparison of the regulatory ratios (WT versus mutant) obtained by overexpression of ScHAP4 or HpHAP4A in ScΔhap4 mutant: genes encoding respiratory chain components. (DOCX) [file pone.0112263.s001.docx]

**Supplementary table S1**: **Comparison of the regulatory ratios (WT versus mutant) obtained by overexpression of *ScHAP4 or HpHAP4-A in Sc∆hap4 mutant:* genes encoding respiratory chain components.**

| Gene | Function | **Fold change *∆hap4** | **Fold change ScHAP4** | **Fold change HAP4A** |
| --- | --- | --- | --- | --- |
|  | **NADH dehydrogenase** |  |  |  |
| *NDI1* | mitochondrial NADH ubiquinone oxidoreductase | 2.63 | 4.82 | 2.41 |
|  | **Complex II** |  |  |  |
| *SDH1* | Flavoprotein subunit of succinate dehydrogenase | 4.17 | 2.3 | 3.49 |
| *SDH2* | Succinate dehydrogenase (ubiquinone) iron-sulfur protein subunit | 3.03 | 6.48 | 3.28 |
| *SDH3* | Succinate dehydrogenase cytochrome b | 1.79 | 4.86 | 2.07 |
| *SDH4* | Succinate dehydrogenase membrane subunit | 2.44 | 4.02 | 2.29 |
|  | **Complex III** |  |  |  |
| *QCR1/COR1* | 44 kDa core protein of yeast coenzyme QH2 cytochrome c reductase | 2.56 | 3.43 | 2.37 |
| *QCR2* | 40 kDa ubiquinol cytochrome c reductase core protein 2 | 2.94 | 3.88 | 1.86** |
| *QCR6* | Ubiquinol-cytochrome c oxidoreductase subunit 6 (17 kDa) | 2.78 | 2.91 | 1.77** |
| *QCR7* | Ubiquinol-cytochrome c oxidoreductase subunit 7 (14 kDa) | 1.75 | 3.44 | 2.66 |
| *QCR8* | Ubiquinol cytochrome-c reductase subunit 8 (11 kDa protein) | 1.47 | 2.73 | 2.21 |
| *QCR9* | 7.3 kDa subunit 9 of the ubiquinol cytochrome c oxidoreductase complex | 1.75 | 2.42 | no |
| *QCR10* | 8.5 kDa subunit of the ubiqunol-cytochrome c oxidoreductase complex | 3.45 | 3.45 | no |
| *RIP1* | Rieske iron-sulfur protein of the mitochondrial cytochrome bc1 complex | 2.13 | 3.53 | no |
|  | **Complex IV** |  |  |  |
| *COX12* | Subunit VIb of cytochrome c oxidase | 2.08 | 2.36 | 2.37 |
| *COX13* | Subunit VIa of cytochrome c oxidase; | 1.96 | 2.25 | 1.44** |
| *COX4* | Subunit IV of cytochrome c oxidase | 2.94 | 3.78 | 1.61 |
| *COX5A* | Cytochrome-c oxidase chain Va | 1.85 | 4.99 | 3.76 |
| *COX5B* | Cytochrome-c oxidase chain Vb | no | no | no |
| *COX6* | Subunit VI of cytochrome c oxidase | 2.78 | 3.73 | 2.27 |
| *COX7* | Subunit VII of cytochrome c oxidase | 1.61 | 3.07 | 2.6* |
| *COX8* | Cytochrome-c oxidase chain VIII | 1.75 | 1.9 | 1.57** |
| *COX9* | Subunit VIIa of cytochrome c oxidase | 1.96 | 2.61 | 1.44** |
|  | **Complex V** |  |  |  |
| *ATP1* | Mitochondrial F1F0-ATPase alpha subunit | 1.49 | 3.12 | 1.95 |
| *ATP2* | Mitochondrial F1F0-ATPase beta subunit | 2.22 | 4.94 | 2.07 |
| *ATP3* | Mitochondrial F1F0-ATPase gamma subunit | 2.17 | 4.74 | 2.72 |
| *ATP4* | Mitochondrial F1F0-ATPase delta subunit | 2.22 | 4.71 | 3.08 |
| *ATP5* | Mitochondrial F1F0-ATPase subunit 5 | 2.78 | 4.44 | 2.37 |
| *ATP7* | Mitochondrial F1F0-ATPase d subunit | 3.22 | 3.88 | 4.1 |
| *ATP14* | Mitochondrial F1F0-ATPase subunit h | 2.78 | 4.68 | 2.45 |
| *ATP15* | Mitochondrial F1F0-ATPase epsilon subunit | 1.58 | 3.57 | 1.48** |
| *ATP16* | Mitochondrial F1F0-ATPase delta subunit | 2.94 | 2.57 | 3.09 |
| *ATP17* | Mitochondrial F1F0-ATPase subunit f | 2.32 | 3.53 | 2.56 |
| *ATP20* | Mitochondrial F1F0-ATPase subunit g | 2.77 | 3.92 | 3.19 |
| *ATP21/TIM11* | Mitochondrial F1F0-ATPase subunit e | 1.92 | 1.97** | 1.74** |

*As a reference, the set of data obtained by Affymetrix data with the deletion of Sc∆hap4 are indicated (data in [[5](#_ENREF_5)]). While these data are not directly comparable (Affymetrix versus Agilent), the preparation of RNAs were done in the same conditions (growth in galactose 2%, OD600 0.6 to 0.8).** p-values between 0.005 and 0.05
