# Supplementary material for: Functional Study of the Hap4-Like Genes Suggests That the Key Regulators of Carbon Metabolism HAP4 and Oxidative Stress Response YAP1 in Yeast Diverged from a Common Ancestor
Source: PLoS One. 2014 Dec 5;9(12):e112263. doi: 10.1371/journal.pone.0112263 (PMC4257542; doi:10.1371/journal.pone.0112263)
Supplement: Table S2 — Comparison of the regulatory ratios (WT versus mutant) obtained by overexpression of ScHAP4 or HpHAP4A in ScΔhap4 mutant: genes encoding TCA cycle and related pathways enzymes. (DOCX) [file pone.0112263.s002.docx]

**Supplementary table 2**: **Comparison of the regulatory ratios (WT versus mutant) obtained by overexpression of *ScHAP4 or HpHAP4A in Sc∆hap4 mutant:* genes encoding the TCA cycle and related pathway enzymes.**

| **Gene** | **Function** | **Fold change *∆hap4** | **Fold change ScHAP4** | **Fold change HAP4A** |
| --- | --- | --- | --- | --- |
| *IDP2* | Cytosolic form of NADP-dependent isocitrate dehydrogenase | 3.7 | 3.49 | 4.64 |
| *PYK2* | Pyruvate kinase; glucose-repressed isoform | 3.22 | no | no |
| *PCK1* | Phosphoenolpyruvate carboxylkinase | 2.94 | no | 6.66 |
| *KGD2* | Component of alpha-ketoglutarate dehydrogenase | 2.94 | 8.67 | no |
| *ACO1* | Mitochondrial aconitase | 2.05 | no | no |
| *FUM1* | Mitochondrial and cytoplasmic fumarase | 2.56 | 3.2 | 4.34 |
| *ICL1* | Isocitrate lyase | 2.56 | 3.29* | 6.94 |
| *KGD1* | Alpha-ketoglutarate dehydrogenase | 2.43 | 4.67 | 2.68 |
| *MLS1* | Carbon-catabolite sensitive malate synthase | 2.43* | 14.21 | 6.31 |
| *MDH1* | Mitochondrial malate dehydrogenase | 2.27 | 3.5 | 7.34 |
| *LPD1* | Dihydrolipoamide dehydrogenase precursor | 2.08 | 2.56 | no |
| *CIT1* | Citrate synthase. Nuclear encoded mitochondrial protein. | 2.04 | 2.54 | 2.2 |
| *PYC1* | Pyruvate carboxylase | 2.04 | no | no |
| *IDP1* | Mitochondrial form of NADP-specific isocitrate dehydrogenase | 1.96 | 2.32 | no |
| *ACS1* | Inducible acetyl-coenzyme A synthetase | 1.96 | no | 2.02 |
| *ALD2* | Aldehyde dehydrogenase; (NAD(P)+) | 1.88 | 4.8 | no |
| *MDH2* | Cytosolic malate dehydrogenase | 1.85 | no | no |
| *IDHI* | Subunit of mitochondrial isocitrate dehydrogenase | 1.72 | 2.42 | no |
| *CIT2* | Non-mitochondrial citrate synthase | 1.53 | no | no |
| *ICL2* | Isocitrate lyase | 1.4 | no | 5.35 |
| *MDH3* | Malate dehydrogenase | 1.26 | no | no |
| *IDH2* | NAD+-dependent isocitrate dehydrogenase | 1.2 | no | no |

* see footnotes of Table S1
