# Supplementary material for: Functional Study of the Hap4-Like Genes Suggests That the Key Regulators of Carbon Metabolism HAP4 and Oxidative Stress Response YAP1 in Yeast Diverged from a Common Ancestor
Source: PLoS One. 2014 Dec 5;9(12):e112263. doi: 10.1371/journal.pone.0112263 (PMC4257542; doi:10.1371/journal.pone.0112263)
Supplement: Table S3 — Comparison of the regulatory ratios (WT versus mutant) obtained by overexpression of ScHAP4 or HpHAP4A in ScΔhap4 mutant: genes encoding components of the mitochondrial translation apparatus. (DOCX) [file pone.0112263.s003.docx]

**Supplementary table 3**: **Comparison of the regulatory ratios (WT versus mutant) obtained by overexpression of *ScHAP4 or HpHAP4A in Sc∆hap4 mutant:* genes encoding components of the mitochondrial translation apparatus**

| **Gene** | **Function** | **Fold change *∆hap4** | **Fold change ScHAP4** | **Fold change HAP4A** |
| --- | --- | --- | --- | --- |
| *MEF2* | Mitochondrial elongation factor G-like protein | 4 | 9.3 | 4.21 |
| *RPM2* | Subunit of mitochondrial RNase P | 3.84 | 4.03 | 5.35 |
| *TUF1* | Mitochondrial translation elongation factor Tu | 2.22 | no | 2.02 |
| *MSD1* | Mitochondrial aspartyl-tRNA synthetase | 2.17 | no | 1.93 |
| *MRPL6* | Mitochondrial ribosomal protein MRPL6 | 1.96 | no | 2.74 |
| *MRPL19* | Mitochondrial ribosomal protein of the large subunit | 1.85 | no | 1.96 |
| *MSR1* | Arginyl-tRNA synthetase | 1.85 | no | no |
| *YMR31* | Mitochondrial ribosomal protein | 1.85 | 3 | no |
| *MRPS5* | Probable mitochondrial ribosomal protein S5 | 1.81 | no | 2.36 |
| *MRP51* | Mitochondrial ribosomal protein (small subunit) | 1.79 | no | 2.67 |
| *MRPL7* | Mitochondrial ribosomal protein MRPL7 | 1.79 | no | 3.03 |
| *MRPL15* | Mitochondrial ribosomal protein MRPL15 | 1.79 | no | 2.75 |
| *RML2* | Mitochondrial ribosomal protein L2 | 1.75 | no | 2.14 |
| *MEF1* | Mitochondrial elongation factor G-like protein | 1.72 | no | 3.63 |
| *ISM1* | Mitochondrial isoleucyl-tRNA synthetase | 1.72* | no | no |
| *PET123* | Mitochondrial ribosomal protein of small subunit | 1.69 | no | 2.59 |
| *MRF1* | Mitochondrial polypeptide chain release factor | 1.69 | no | 2.26 |
| *MRPL11* | Mitochondrial ribosomal protein MRPL11 | 1.69 | no | 2.36 |
| *MST1* | Mitochondrial threonine-tRNA synthetase | 1.67 | no | no |
| *MSE1* | Mitochondrial glutamyl-tRNA synthetase | 1.67 | no | no |
| *SLS1* | coupling of mitochondrial translation and transcription | 1.67* | no | no |
| *MRPS17* | weak similarity to bacterial ribosomal protein S17 | 1.64 | no | 2.59 |
| *GRS2* | strong similarity to glycyl-tRNA synthetases | 1.64 | no | no |
| *MSM1* | Mitochondrial methionyl-tRNA synthetase | 1.61 | no | 1.9 |
| *NAM9* | putative Mitochondrial S4 ribosomal protein | 1.61 | no | 2.68 |
| *AIM10* | similarity to *E.coli* prolyl-tRNA synthetase | 1.62 | no | no |
| *MRP4* | Mitochondrial ribosomal protein homologous to *E. coli* S2 | 1.56 | no | no |
| *MRPL40* | Mitochondrial ribosomal protein MRPL40 | 1.56 | no | 3.93 |
| *MRPL9* | Mitochondrial ribosomal protein MRPL9 | 1.56 | no | 2.29 |
| *MRPL35* | Mitochondrial ribosomal protein MRPL35 | 1.56 | 1.97 | 2.9 |
| *MRPL10* | Mitochondrial ribosomal protein MRPL10 | 1.53 | no | 1.99 |
| *MRPL17* | ribosomal protein of the large subunit (YmL30) | 1.51 | no | 2.82 |
| *NAM2* | Mitochondrial leucyl tRNA synthetase | 1.51 | no | 2.08 |
| *YMR158W* | Weak similarity to *E.coli* ribosomal S8 protein | 1.51 | no | 3.24 |

* see footnotes of Table S1
