# Supplementary material for: Functional Study of the Hap4-Like Genes Suggests That the Key Regulators of Carbon Metabolism HAP4 and Oxidative Stress Response YAP1 in Yeast Diverged from a Common Ancestor
Source: PLoS One. 2014 Dec 5;9(12):e112263. doi: 10.1371/journal.pone.0112263 (PMC4257542; doi:10.1371/journal.pone.0112263)
Supplement: Table S4 — List of S. cerevisiae genes upregulated in presence of H202 when ScYAP1 or HpHAP4-B were overexpressed in ScΔyap1 mutant. (DOCX) [file pone.0112263.s004.docx]

**Supplementary table 4: *S. cerevisiae* genes upregulated in presence of H_2_0_2_ when *ScYAP1* or *HpHAP4-B* were overexpressed in *Sc∆yap1* mutant**

| **Process** | ***YAP1*** | ***YAP1* and *HpHAP4-B*** | ***HpHAP4-B*** |
| --- | --- | --- | --- |
| Iron deficit | *AFT2, TIS11* |  | *RGI1* |
| Metal homeostasis | *ARR3, CCC2, MMT1, ISU2, ISU1, FRE3*, FRE1, FRE5, ARN2*, RCS*1, ARN1, FET3, ZRT1, SIT1, COT*1, FMP23, YCR102C, MRS4, IBA57, DRE2, FIT2, FIT3* | *CUP1_2, CUP1_1* | *YFH*1* |
| Stress response | *MDJ1, YAP7*, MCX1*, NRG1,YER130C, SNG1, YGR250C, QDR2, DAK2, FLR1* | *HSP30, HSP26, HSP33, HSP104, HSP82, CPR5, YGP1, HOR2, YAP6, HSP78, HSP42, SSA4, STI1*, SSE2, SPG4, DDR48, TPO2, CPR5, CPR6, DAK1, HSP31, YNL234W, SMP1** | *UBI4, TSL1, TPS2, TPS1, NTH1, GRE1, GRE3, DDR2, SIS1, CIN5, FUN19, GAC1, NTH1, NVJ1, MRK1, SSA3, RLM1, RPN4, SDP1, SIP18, SIS1, STF2, SYM1, UGX2, YPK2, FRT2, FMP16, FMP43,YDR034W-B, DCS2, YPL088W, ASR1, SPI1* |
| Oxidative stress, oxidation/reduction process | *YAP1, TRX2, TRX1*, LYS7, PEX21, OYE2, LIF1*, THI4, ADH6, AAD6, AAD4, AAD3, AAD10, AAD16, FDH1, FDH2, ALD5, CTA1, BNA1, SFA1, SOD2 (YHR008C), YKL071W, YLR460C, NCE103, ROX1, AFT2, TTR1, FRM2* | *TSA2, BTN2, OYE3, UGA2*, CTA1*, SOD2*, GTT2, ALD5, AHP1, SNO4, HSP32, GND2, RCK1, YDL124W, JLP1, CUP1_2, CUP1_1, EMP46, PRX1, HBN1, YML131W, GOR1, ZTA1, ERO1** | *HSP2, HSP12, GRX1, CTT1, ALD3, ALD4, GAD1, NDE2, OM45*, GDH3, GPX1, PNC1, RNY1, BDH2, AIM17, YKL151C, FMP46* |
| Glutathione metabolism | *GSH1, GPX2, GTO3, GLR1, YCF1* | *ECM4, GTT2,* | *GPX1* |
| Drugs-resistance | *PDR16*, ATR1, PDR12* | *JIP4, YHK8* |  |
| DNA repair, replication stress response | *LIF1, NTG1** | *DDR48, GSP2, PRX1, HBN1, HSP31, YLR108C, YML131W, YNL134C, GOR1, ESC2* | *PHR1, CYC7, GDB1, EMI2, GCY1, OM45*, LSB1, MBF1*, YKU70, SIS1, SOL4, SRL3, STF2, TFS1, RAD34* |
| Riboflavin | *RIB1, MCH5* |  |  |
| B6 | *SNO1, SNZ1* |  |  |
| Biotin | *BIO3, BIO4* |  |  |
| Aminoacid metabolism and transport | *CPA2, ARG4, ARG1, AAR2, ARR3, HIS3, HIS4, HIS5, HIS7, LEU1, LEU4, BAT1, ASN*1, ARG8*, ARG5, ARG6, ARG2*, UGA3, ARO3, BAP2, BAT, TMT1, HOM2, ILV2, LYS20* | *BAP2*, SRY1*, AGP3, BTN2* | *GAD1, CAT2, PDC6* |
| Cell-cycle, signalling, sporulation | *UBC11, SPC2, CTF13, MCM*1, TEA1, MSH4, SYF2, SPO21, PKH2, MUM3, MF(ALPHA)2* | *PRR2, PCL5, REC8* | *SPO13, GAC1, UGP1, YAK1, TPK1, YPK2, SKM1, BAG7, MEK1, AFR1, SGA1, AMA1, CSM4, TOS8, EMI2, SIC1, SDS22, SPR3, SRL3, MF(ALPHA)1, MSC1, VHS1, OSW2* |
| Carbohydrate metabolism | *PYC1*, MLS1, LYS20, ICL1, ICL2, STD1, TYE7* | *HXT4, HXT1*, HXT3, MIG2, GND2, ERR1, ERR2, ERR3, HOR2, YNL234W, GOR1, REG2, VID24, ICY2** | *ACA1, GPM2, FBP26, MTH1, HXT5, HXT17*, HXT8, HXT7, HXT6, HXT13, HXT15, HXT16, HXK1, GDB1, PGM2, GSY1, GSY2, GLG1, PCL10, GLC3, GLK1, GPH1, CAT2, FYV10, GAC1, GCY1, MAL12, MAL31, MAL32, MIG1, GIP2, PFK26, PDC6, PIG2, RMD5, SOL4, SOR1, SPI1, SYM1, TKL2, UGP1, XKS1, YAK1, SOR2, NQM1, YJR096W, YLR345W, YNR073C, YIG1, ASR1** |
| Aerobic growth, respiration, mitochondria | *MLS1, FMP23, NCA3, OAC1, ROX1, bI3, IBA57, YAT2* |  | *COX5B, CYC7, CYB2, GDH3, IKS1, ICF1, SPI1, UTR5, RGI1, FMP33, USV1, STF2* |
| Gluconeogenesis |  | *SIP4** | *CAT8, ENO1, FBP26, GPM2, NDE2* |
| Fatty acid ß-oxidation, peroxisomes | *PEX21* |  | *POT1, POX1, IDP3, ECI1, PXA1, PXA2, PCD1, PEX18* |
| Autophagy, proteolytic catabolism | *APE1* | *ROG3* | *PAI3, APE3, GYP7, UBC8, UBP11, APG16, AUT1, PEX18, RPN4, AUT7, FYV10, MRK1, RMD5, RPN4, UBX3, ATG29, UIP4* |
| Sterol synthesis, transport | *ERG27* | *BTN2, PRY1, SET6* | *SKM1* |
| Stationary phase growth |  | *MOH1, SPG4, YGP1* | *SPG1, TFS1, GUD1* |
| Cell wall | *ECM13, ECM34, ECM40, KTR5, PKH2* |  | *YLR194C, PIR3* |

* indicates that the p-value is not as good as the one indicated in Material and Methods (<0.5%) but

only <5%.
